# Ex Vivo Blockade of the PD-1 Pathway Improves Recall IFNγ Responses of HIV-Infected Persons on Antiretroviral Therapy

**DOI:** 10.3390/vaccines11020211

**Published:** 2023-01-18

**Authors:** Natalie Fischhaber, Moritz Schmiedeberg, Sabrina Kübel, Ellen G. Harrer, Thomas Harrer, Krystelle Nganou-Makamdop

**Affiliations:** 1 Institute of Clinical and Molecular Virology, Universitätsklinikum Erlangen, Friedrich-Alexander Universität Erlangen-Nürnberg, Schlossgarten 4, 91054 Erlangen, Germany; 2 Infectious Diseases and Immunodeficiency Section, Department of Internal Medicine 3, Universitätsklinikum Erlangen, Friedrich-Alexander-Universität Erlangen-Nürnberg, 91054 Erlangen, Germany

**Keywords:** T cell exhaustion, PD-1 pathway, HIV infection, vaccine-induced immunity, anti-PD-1, anti-PD-L1

## Abstract

Despite antiretroviral therapy (ART), immune exhaustion persists in HIV infection and limits T cell responses to HIV or other pathogens. Moreover, HIV infection results in the loss of pre-existing immunity. Here, we investigated the effect of blocking the PD-1 pathway on recall IFNγ responses to tetanus toxoid (TT) and measles virus (MV) antigens in HIV-infected persons on ART with prior TT and MV immunity. The ex vivo treatment of lymphocytes with anti-PD-1 and anti-PD-L1 antibodies significantly increased TT- and MV-specific IFNγ responses. The responses to TT and MV antigens alone or in combination with antibodies blocking the PD-1 pathway positively correlated with CD4 T cell levels. Furthermore, T cell PD-1 expression levels inversely correlated with recall IFNγ responses in combination with antibodies blocking the PD-1 pathway but not with IFNγ responses to antigens only. Our study suggested that targeting the PD-1 pathway may boost vaccine-induced pre-existing immunity in HIV-infected persons on ART depending on the degree of immune exhaustion.

## 1. Introduction

Immune cell exhaustion is a hallmark of chronic viral infections. Continuous antigenic exposure drives the permanent activation of T cells, which, in turn, leads to a gradual loss of effector functions and the increased expression of inhibitory receptors, such as programmed death 1 (PD-1) [1]. Exhausted CD4 and CD8 T cells exhibit reduced production of cytokines, such as IL-2, TNFα and IFNγ [1,2], that are essential for their respective helper and cytolytic functions. In untreated human immunodeficiency virus (HIV) infection, PD-1 expression is increased on both CD4 and CD8 T cells and correlates with impaired HIV-specific responses. Moreover, a blockade of the PD-1 pathway was shown to increase proliferation and IFNγ responses of HIV-specific CD4 and CD8 T cells [3,4]. To date, antiretroviral therapy (ART) allows for the control of HIV infection by reducing productively infected cells and maintaining HIV plasma levels below the limit of detection in most treated individuals [5,6]. However, even after years of ART, latently infected memory CD4 T cells persist and treatment interruption is followed by a rapid virus rebound. Various studies showed that immune cell activation and T cell exhaustion decrease under ART but remain higher compared with levels of HIV-uninfected persons [7,8,9]. Furthermore, PD-1 expression was found to be associated with incomplete immune reconstitution under ART [10] and expression levels of the inhibitory receptors PD-1, T cell immunoglobulin and mucin domain-containing protein 3 (TIM3), along with lymphocyte-activation gene 3 (LAG3), were reported to be associated with time to viral rebound following ART interruption [11]. Clearly, T cell exhaustion contributes to immune dysfunction in ART-treated HIV infection.

Antigen-specific T cell responses are essential to the maintenance of immunological memory. Recently, it was shown that HIV-infected persons on ART display lower vaccinia-virus-specific IFNγ and TNF responses of CD4 T cells, denoting loss of pre-existing immunity to the vaccinia virus [12]. Likewise, the loss of antigen-specific CD4 T cell responses was shown for mumps, influenza [13], the vaccinia virus and mycobacterium tuberculosis [14]. While the initiation of ART was found to increase the proportion of HIV-infected persons with recall CD4 T cell responses to tetanus toxoid (TT) [15] or to transiently increase lymphocyte proliferative responses to TT [16], others reported lower proliferative capacity of TT-specific CD4 T cells in HIV-infected persons compared with uninfected persons [17]. Thus, HIV infection impairs recall responses to prior antigens but the role of the immunoregulatory PD-1/PD-L1 pathway in this relationship remains unclear. Here, we investigated the effect of blocking the PD-1 pathway on recall IFNγ responses of HIV-infected persons on ART. In vitro responses to TT and measles virus (MV) antigens were assessed in combination with anti-PD-1 or anti-PD-L1 antibodies in persons with prior TT and MV exposure. Moreover, the relationship between recall IFNγ responses and CD4 T cell counts or PD-1 expression levels on T cells was evaluated.

## 2. Materials and Methods

### 2.1. Study Participants

HIV-infected persons on antiretroviral therapy (ART) were recruited from the Erlangen HIV cohort at the Universitätsklinikum in Erlangen. HIV-uninfected persons were recruited through the Institute of Clinical and Molecular Virology of the Universitätsklinikum in Erlangen. For the comparison of the recall TT responses between HIV-infected and HIV-uninfected groups, individuals with self-reported prior vaccination were recruited. These were 14 HIV-uninfected persons with a median age of 49 years (interquartile range (IQR) 44–54) and 33 HIV-infected persons with a median age of 51 years (IQR 40–54) and a median CD4 T cell count of 647 cells/mm^3^ (IQR 538–794). To assess the effect of anti-PD-1 and anti-PD-L1 antibodies on the recall IFNγ responses of HIV-infected persons on ART, lymphocytes of 30 HIV-infected TT-seropositive and 29 HIV-infected measles virus (MV)-seropositive subjects were probed. Detailed characteristics of the 30 TT- and 29 MV-seropositive participants are presented in Table 1.

The study was approved by the ethics committees of the Universitätsklinikum Erlangen (numbers 235-18B and 250-15B) and carried out in compliance with institutional guidelines. All participants gave written informed consent.

### 2.2. Blood Samples

Whole blood that was freshly collected in citrate tubes (Sarstedt) was immediately processed via centrifugation for the separation of plasma frozen at −80 °C for subsequent assays. Peripheral blood mononuclear cells (PBMCs) were isolated via density centrifugation using Ficoll-Paque Plus (GE Healthcare) and Leukosep Centrifuge Tubes (Greiner Bio-One) prior to cryopreservation in liquid nitrogen.

### 2.3. Anti-Tetanus Antibody Levels

High-binding 96-well Lumitrac plates were coated overnight with tetanus toxoid (0.4 IU/mL). Plates were washed 3 times with PBS-0.05% Tween20 and blocked for 2 h with 5% skimmed milk. Following 3 wash steps with PBS-0.05% Tween20, plasma samples diluted in PBS-0.05% Tween20 and 2% skimmed milk were incubated for 2 h. Plates were then washed 3 times with PBS-0.05% Tween20 prior to the addition of the secondary antibody Goat-anti-human IgG-HRP (Jackson Immunoresearch). Following 1 h of incubation, plates were washed 3 times with PBS-0.05% Tween20, then twice with PBS prior to the addition of ECL solution and measurement of the relative light units per second (RLU/s) with an Orion microplate luminometer (Berthold Detection Systems GmbH).

### 2.4. Measurement of PD-1 Expression Levels on T Cells

Cryopreserved PBMCs were thawed and washed in R10 medium (RPMI1640 supplemented with 10% fetal bovine serum, 2 mM L-glutamine and 100 U/mL penicillin/streptomycin). PBMCs were stained with Fixable viability Dye eFluor 780 (Thermofisher), CD3-FITC (Biolegend, clone OKT3) and PD-1-BV421 (Biolegend, clone EH12.2H7). Following fixation with 4% PFA, stained cells were acquired on an LSR-II cytometer (BD) and data were analyzed on Flow-Jo version 10.

### 2.5. Measurement of Recall IFNγ Responses to Measles Virus and Tetanus Toxin

For a comparison of the recall IFNγ responses to TT between HIV-infected and HIV-uninfected persons, cryopreserved PBMCs were thawed, washed in R10 medium and rested for 6 h at 37 °C. Following the seeding of 1 × 10^6^ cells per well, PBMCs in R10 were either left untreated or stimulated with TT protein (Tetanol, GSK—0.5 I.E) and incubated at 37 °C in 5% CO_2_ for 3 days. To assess the effect of anti-PD-1 and anti-PD-L1 on recall IFNγ responses, cryopreserved PBMCs were thawed, washed in R10 medium and rested overnight at 37 °C. Cells were washed, seeded in 96-well round bottom plates at 0.9 × 10^6^ cells per well in R10 medium and incubated for 6 h with 20 µg/mL or 40 µg/mL anti-PD-1 (Nivolumab), anti-PD-L1 (Durvalumab), or their respective IgG4 and IgG1 isotype controls (BioXCell). Next, TT protein (Tetanol, GSK—0.5 I.E) or an MV 20-mer overlapping peptide pool corresponding to the measles virus hemagglutinin and fusion proteins (EMC, 10 µg/mL) was added per well. As a positive control, PBMCs were stimulated with Staphylococcal enterotoxin B (SEB, Sigma, 0.25 µg/mL final concentration). As negative controls, PBMCs were either unstimulated (TT control) or supplemented with DMSO (MV peptide pool control). Following the addition of all stimuli, cells were incubated at 37 °C in 5% CO_2_ for 3 days. All cell culture supernatants were collected after 3 days of stimulation for the measurement of IFNγ concentrations using the human IFNγ Duoset (R&D) as per the manufacturer’s recommendation. Measurement of IFNγ concentrations was performed in duplicate and the average of the duplicates was reported.

### 2.6. Statistical Analysis

Statistical analyses as indicated in figure legends were performed with the GraphPad Prism version 10 software (GraphPad Software Inc.).

## 3. Results

**Figure 1:**
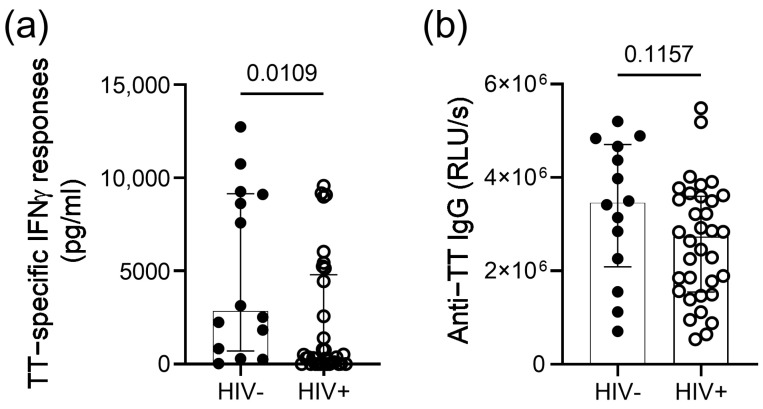
TT-specific IFNγ responses and antibody levels among the HIV-infected and HIV-uninfected study participants. (**a**) IFNγ concentrations in the supernatant 3 days after stimulation with TT protein in persons with self-reported prior vaccination. Background responses (unstimulated condition) are subtracted for each participant. (**b**) Plasma levels of the anti-TT IgG antibodies in persons with self-reported prior vaccination. Medians, interquartile ranges and *p*-values of the comparison between groups using the Mann–Whitney test are presented.

We first compared TT-specific IFNγ responses of 33 HIV-infected persons on ART to responses of 14 HIV-uninfected individuals with self-reported prior TT vaccination. The assessment of recall IFNγ responses to TT in these participants revealed significantly lower IFNγ responses in the group of HIV-infected participants (*p* = 0.01; Figure 1a) despite similar plasma levels of anti-TT IgG antibodies (*p* = 0.11; Figure 1b).

**Figure 2:**
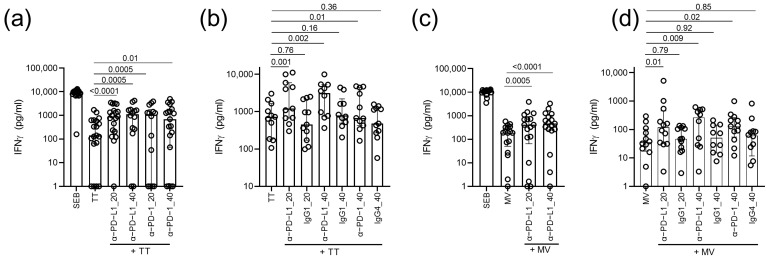
IFNγ responses upon ex vivo blockades of the PD-1 pathway. (**a**) TT-specific IFNγ responses of the HIV-infected TT-seropositive persons upon the addition of anti-PD-1 or anti-PD-L1 antibodies at concentrations of 20 or 40 µg/mL. (**b**) TT-specific IFNγ responses of a separate set of the HIV-infected TT-seropositive persons upon the addition of anti-PD-L1 (20 and 40 µg/mL) or anti-PD-1 (40 µg/mL) and their respective isotype controls, namely, human IgG1 and human IgG4. (**c**) MV-specific IFNγ responses of the HIV-infected MV-seropositive persons upon the addition of anti-PD-L1 at concentrations of 20 or 40 µg/mL. (**d**) MV-specific IFNγ responses of a separate set of HIV-infected MV-seropositive persons upon the addition of anti-PD-L1 (20 and 40 µg/mL) or anti-PD-1 (40 µg/mL) and their respective isotype controls human IgG1 and human IgG4. The background responses (unstimulated condition) were subtracted for each participant. Medians, interquartile ranges and *p*-values of the comparison between stimulation conditions using the Wilcoxon test are presented.

Next, we investigated whether treatment with the anti-PD-1 or anti-PD-L1 antibodies could improve the recall IFNγ responses to TT in HIV-infected persons with prior vaccination. To this end, we first stimulated PBMCs of 19 TT-seropositive HIV-infected persons with TT in the presence of anti-PD-1 or anti-PD-L1 antibodies at final concentrations of 20 or 40 µg/mL. The measurement of IFNγ concentrations after stimulation revealed significantly increased recall IFNγ responses to TT upon the addition of both 20 and 40 µg/mL anti-PD-L1 (*p* ≤ 0.005) and anti-PD-1 (*p* ≤ 0.01; Figure 2a). These findings were re-iterated in a second set of 11 TT-seropositive HIV-infected participants with elevated IFNγ responses to TT that were observed upon the addition of 20 µg/mL or 40 µg/mL anti-PD-L1 (*p* ≤ 0.002; Figure 2b) and 40 µg/mL anti-PD-1 antibodies (*p* = 0.01; Figure 2b), while no effect was observed upon treatment with respective isotype controls (*p* ≥ 0.16; Figure 2b). These data demonstrated that in vitro IFNγ recall responses to TT could be improved by blockade of the PD-1 axis using anti-PD-1 or anti-PD-L1 antibodies.

To assess whether our findings could be replicated for a different antigen, we stimulated the PBMCs of 17 MV-seropositive HIV-infected persons with MV antigens in presence of anti-PD-L1 at final concentrations of 20 or 40 µg/mL. Similar to TT, treatment with anti-PD-L1 antibodies resulted in a significant increase in the IFNγ responses to MV antigens (*p* ≤ 0.005; Figure 2c). In a second set of 12 MV-seropositive HIV-infected participants, increased IFNγ responses to MV were observed upon the addition of 20 µg/mL or 40 µg/mL anti-PD-L1 (*p* ≤ 0.01; Figure 2d) and 40 µg/mL anti-PD-1 antibodies (*p* = 0.02; Figure 2d), whereas no effect was observed upon treatment with respective isotype controls (*p* ≥ 0.79; Figure 2d).

Given that HIV infection causes the depletion of CD4 T cells and that CD4 T cell recovery under ART varies between persons, we next explored the relationship between CD4 T cell counts and recall IFNγ responses in the presence of antibodies blocking the PD-1 pathway. In the 30 TT-seropositive and 29 MV-seropositive HIV-infected persons on ART, the percentages of CD4 T cells and the absolute CD4 T cell counts correlated with IFNγ responses to TT alone or in the presence of anti-PD-1 or anti-PD-L1 antibodies (Table 2). MV-specific IFNγ responses in the presence of anti-PD-1 or anti-PD-L1 antibodies but not responses to MV alone also correlated with the percentages and absolute counts of CD4 T cells. In addition, IFNγ responses to MV antigens correlated with the CD4:CD8 T cell ratio. Overall, these data demonstrated that irrespective of the targeting of the PD-1 pathway, higher IFNγ responses could generally be observed in participants with higher CD4 T cell levels.

**Figure 3:**
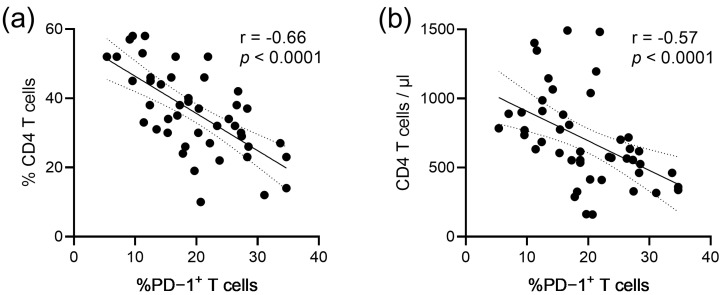
Correlation between the blood CD4 T cell levels and T cell PD-1 expression levels. (**a**) Linear regression analysis with percentages of CD4 T cells. (**b**) Linear regression analysis with CD4 T cell counts (cells/µL). The 95% confidence interval of linear regression, as well as the rhos and *p*-values of the Spearman correlations, are presented.

Of note, we observed in these participants an inverse correlation between the frequencies of PD-1+ T cells and both the percentages of CD4 T cells (r = −0.66; *p* < 0.0001; Figure 3a), as well as the absolute CD4 T cell counts (r = −0.57; *p* > 0.0001; Figure 3b).

**Figure 4:**
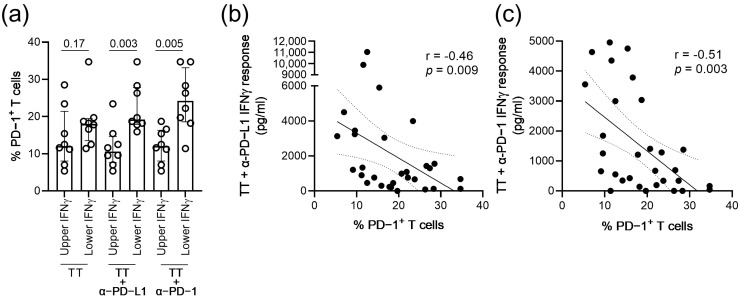
Association between the T cell PD-1 expression levels and TT-specific IFNγ responses in the presence of antibodies blocking the PD-1 pathway. (**a**) T cell PD-1 expression levels of the participants with upper or lower quartiles of IFNγ responses to TT alone, TT+ anti-PD-L1 (20 µg/mL) or TT+ anti-PD-1 (40 µg/mL). Medians, interquartile ranges and *p*-values of the comparison between groups using the Mann–Whitney test are presented. (**b**) Correlation analysis between the T cell PD-1 expression levels and IFNγ responses to TT+ anti-PD-L1 (20 µg/mL) of the 30 TT-seropositive HIV-infected persons. (**c**) Correlation analysis between the T cell PD-1 expression levels and IFNγ responses to TT+ anti-PD-1 (40 µg/mL). The 95% confidence interval of the linear regression, as well as the rhos and *p*-values of the Spearman correlations, are presented.

To determine whether the improvement in recall IFNγ responses upon the addition of antibodies blocking the PD-1 pathway was associated with PD-1 expression levels, the frequencies of PD-1+ T cells were compared between the upper and lower quartiles of IFNγ responses of the 30 TT-seropositive ART-treated HIV-infected persons. Among the TT-seropositive HIV-infected participants, the ex vivo levels of PD-1+ T cells were significantly higher in participants with the lowest TT-specific recall IFNγ responses in the presence of anti-PD-L1 (*p* = 0.003; Figure 4a) or anti-PD-1 (*p* = 0.005; Figure 4a). No significant difference was observed between the T cell PD-1 expression levels and IFNγ responses to TT only (*p* = 0.17; Figure 4a).

Throughout the entire cohort of 30 TT-seropositive ART-treated HIV-infected persons, the ex vivo levels of PD-1+ T cells inversely correlated with the IFNγ responses in the presence of anti-PD-L1 (r = −0.46; *p* = 0.009; Figure 4c) or anti-PD-1 (r = −0.51; *p* = 0.003; Figure 4d).

**Figure 5:**
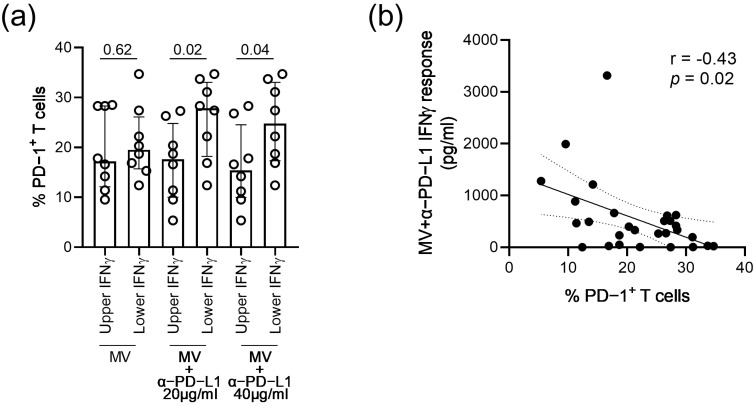
Association between the T cell PD-1 expression levels and MV-specific IFNγ responses in the presence of antibodies blocking the PD-1 pathway. (**a**) T cell PD-1 expression levels of the participants with upper or lower quartiles of IFNγ responses to MV alone, MV+ anti-PD-L1 (20 µg/mL) or MV+ anti-PD-L1 (40 µg/mL). Medians, interquartile ranges and *p*-values of the comparison between groups using the Mann–Whitney test are presented. (**b**) Correlation analysis between the T cell PD-1 expression levels and IFNγ responses to TT+ anti-PD-L1 (40 µg/mL) of the 29 MV-seropositive HIV-infected persons. The 95% confidence interval of the linear regression, as well as the rhos and *p*-value of the Spearman correlations, are presented.

Similar to observations made in the TT-seropositive participants, ex vivo levels of PD-1+ T cells were significantly higher in participants with the lowest MV-specific recall IFNγ responses in the presence of anti-PD-L1 (*p* ≤ 0.04; Figure 5a), while no significant difference was observed between the T cell PD-1 expression levels and IFNγ responses to MV only (*p* = 0.64; Figure 5a). Likewise, the frequencies of PD-1+ T cells among the 29 MV-seropositive HIV-infected participants were inversely associated with IFNγ responses in the presence of anti-PD-L1 (r = −0.43; *p* = 0.02; Figure 5b). Altogether, these data suggested that the magnitude of the IFNγ recall responses upon a blockade of the PD-1 pathway was influenced by the degree of immune exhaustion in HIV-infected persons on ART.

## 4. Discussion

The loss of pre-existing immunity is commonly reported in HIV infection, during which T cell exhaustion persists despite ART. However, there is limited knowledge of the effect of targeting the PD-1 pathway on recall responses in ART-treated HIV infection. Here, we showed that blocking the PD-1 pathway using anti-PD-1 or anti-PD-L1 antibodies improved recall IFNγ responses to TT and MV in HIV-infected persons on ART.

Targeting of the PD-1 pathway was shown to improve HIV-specific responses of HIV-infected persons and, therefore, is being considered an attractive approach to empower HIV cure strategies [3,4,18]. We showed here that blocking the PD-1 pathway using anti-PD-1 or anti-PD-L1 antibodies may provide an additional benefit, namely, that of improving recall immune responses to other pathogens in HIV-infected persons on ART. The loss of pre-existing immunity in HIV infection highlights the need for improvement strategies. Because vaccination and booster immunizations are commonly reported to show lower efficacy in HIV-infected persons [19,20], additional exposure to the antigen only may not be sufficient to rescue antigen-specific immunity. With at least two-thirds of study participants showing increased recall IFNγ responses to antigens upon ex vivo treatment with anti-PD-1 or anti-PD-L1 antibodies, our findings suggested that antibodies targeting the PD-1 pathway may be considered as adjuvants to boost vaccine-induced immunity in HIV infection.

Various studies showed that T cell exhaustion during chronic viral infections can be reversed by blocking the PD-1 pathway. For instance, anti-PD-L1 was shown to decrease the virus load and increase CD8 T cell IFNγ responses, as well as survival in chronic LCMV infection [21]. Likewise, the administration of anti-PD-1 antibodies in HCV-infected chimpanzees resulted in a reduced virus load and increased IFNγ responses of lymphocytes [22]. In addition, previous studies reported an inverse correlation between PD-1 expression on CD4 T cells and the CD4 T cell count in untreated HIV infection [4]. In our study on ART-treated HIV-infected persons, the CD4 T cell counts were inversely correlated with T cell PD-1 expression. These, in turn, were inversely correlated with the recall IFNγ responses in the presence of anti-PD-1 or anti-PD-L1 antibodies, while the CD4 T cell count was positively correlated with these recall IFNγ responses. Given that ongoing T cell activation is associated with incomplete CD4 T cell recovery under ART [23] and persistent T cell activation drives immune exhaustion, it is plausible that incomplete CD4 T cell reconstitution, T cell activation and T cell exhaustion all invigorate one another. Importantly, our findings suggested that both the CD4 T cell count and the expression of PD-1 on T cells may serve as predictors of the response to treatment with anti-PD-1 or anti-PD-L1 antibodies. Although PD-1 is often referred to as an inhibitory receptor, its biological function on T cells is to help prevent excessive T cell signaling. During the interaction between T cells expressing PD-1 and antigen-presenting cells expressing PD-L1 and PD-L2, PD-1 binds to its ligands to transmit a negative costimulatory signal, leading to reduced TCR signaling [24]. As such, the mechanisms of action of PD-1 and PD-L1 antibodies involve the normalization of costimulation and TCR signaling. Studies on chronic viral infections showed that long-term reversion of the PD-1/PD-L1 signaling using antibody treatment is inhibited via epigenetic reprogramming of the exhausted T cells [25,26]. Longitudinal studies with the administration of anti-PD-1 or anti-PD-L1 antibodies at the time of vaccination would provide more insight into how targeting the PD-1 pathway affects the induction and longevity of vaccine-induced immunity, which is known to decay more rapidly in HIV infection. While our study participants did not reveal an association between age and PD-1 expression, future studies on targeting the PD-1 pathway to boost vaccine-induced immunity may include various age groups and assess the influence of age that was shown to increase PD-1 expression [27].

It is important to acknowledge that the numerous antibodies that target the PD-1 pathway vary in their efficacy. For one, half-maximal effective concentrations were found to be higher for the anti-PD-1 antibodies nivolumab and pembrolizumab compared with the anti-PD-L1 antibodies atezolizumab, avelumab and durvalumab [28]. A major factor that may account for this difference between anti-PD-1 and anti-PD-L1 antibodies is that next to binding to PD-1, PD-L1 also binds to CD80, which is an important costimulatory molecule in the interaction between T cells and antigen-presenting cells [29]. Thus, by preventing the binding of PD-L1 to both PD-1 and CD80, anti-PD-L1 antibodies block two distinct pathways, resulting in enhanced T cell responses. In our study, 40 µg/mL anti-PD-L1 induced higher IFNγ responses to TT compared with 40 µg/mL anti-PD-1 but a similar observation was not made for responses to MV. With regard to the use of anti-PD-1/PD-L1 as adjuvants, a difference in efficacy may be influenced by various factors, such as the type of antigen and underlying mechanisms of protective responses. Moreover, several pathways aside from the PD-1/PD-L1 axis are involved in the establishment of T cell exhaustion and include the expression of other molecules, such as cytotoxic T-lymphocyte antigen-4 (CTLA-4), TIM-3, LAG-3 and T cell immunoreceptor with immunoglobulin and ITIM domains (TIGIT) that all regulate T cell function through independent mechanisms [30]. Thus, the combination of antibodies to target different T cell exhaustion pathways may be more effective than the use of individual antibody treatment. For example, the combined blockade of LAG-3 and PD-L1 during chronic LCMV infection was shown to be more effective at reducing viral loads than the blockade of LAG-3 or PD-L1 alone [31]. Likewise, the combined blockade of both TIGIT and PDL-1 was superior at restoring the proliferative capacity of HIV-specific CD8 T cells compared with either single blockade [32]. It may be worth exploring whether similar principles of combinational targeting of different T cell exhaustion pathways are the most beneficial for boosting antigen-specific responses during vaccination or booster administrations in HIV-infected persons. In cancer, the use of immune checkpoint inhibitors is costly [33] and may result in adverse effects [34]. Given the lack of safety data on the use of immune checkpoint inhibitors in HIV-infected noncancer participants, clinical studies with this demographic group should evaluate the adverse effects and cost of treatment. Ongoing and future clinical studies on the administration of low doses of immune checkpoint inhibitors to reverse HIV latency may provide an opportunity to assess both the toxicity and the effect on recall responses to prior immunity.

A limitation of our study was that it did not address whether the blockade of the PD-1 pathway improved the IFNγ responses of CD4 T cells or CD8 T cells, which both express high PD-1 levels in HIV infection. The delineation of cytokine responses at a cell subset level requires approaches, such as flow cytometry, that have a higher resolution compared with ELISA. However, prior immunity in our study participants was established more than 10 years to several decades ago. Here, we opted for an ELISA-based readout given its higher sensitivity, which allowed for the detection of recall IFNγ responses long after antigenic exposure in as many participants as possible, thereby facilitating an adequate correlation analysis with T cell PD-1 levels. A more granular evaluation of T cell responses at a cell subset level may be considered in future studies involving in vivo antigenic exposure, such as infection or revaccination, that may expand responses to more easily detectable ranges. Moreover, we did not assess the effect of blocking the PD-1 pathway on recall responses of HIV-uninfected persons. Therefore, we could not exclude the possibility that targeting the PD-1 pathway could boost recall IFNγ responses of HIV-uninfected persons, particularly those with factors that are known to induce T cell exhaustion, such as viral hepatitis [35] or aging [27].

To conclude, our findings suggested that targeting the PD-1/PD-L1 pathway boosts antigen-specific responses to pathogens other than HIV in infected persons on ART, and may be considered as a strategy to prevent the loss of vaccine-induced immunity in HIV infection.

## Figures and Tables

**Table 1:** Characteristics of TT- and MV-seropositive ART-treated HIV-infected study participants.

	TT Seropositive (n = 30)	MV Seropositive (n = 29)
Age, median years (IQR)	50 (40–56)	49 (41–55)
Male-to-female ratio	19:11	17:12
CD4 T cell count, median cells/µL (IQR)	676 (423–897)	672 (462–772)
CD4:CD8 T cell ratio, median (IQR)	1.9 (1.2–2.6)	1.3 (1.1–1.4)
Plasma virus load, median copies/mL (IQR)	<20 (<20–<20)	<20 (<20–<20)

IQR: interquartile range; TT: tetanus toxoid; MV: measles virus.

**Table 2:** Correlation analysis between blood T cell levels and recall IFNγ responses.

IFNγ Responses	CD4 T Cell Count *Rho; p*	% CD4 T Cells *Rho; p*	CD4:CD8 Ratio *Rho; p*
TT only	0.43; 0.01	0.41; 0.02	−0.06; 0.76
TT + αPD-L1 20 µg/mL	0.50; 0.005	0.49; 0.005	−0.06; 0.76
TT + αPD-1 40 µg/mL	0.54; 0.001	0.55; 0.001	−0.15; 0.42
MV only	0.24; 0.21	0.26; 0.18	0.42; 0.02
MV + αPD-L1 20 µg/mL	0.49; 0.006	0.41; 0.03	0.41; 0.02
MV + αPD-L1 40 µg/mL	0.46; 0.02	0.48; 0.01	0.45; 0.02

## Data Availability

The data presented in this study are available on request from the corresponding author.

## References

[1] Wherry E.J. (2011). T Cell Exhaustion. Nat. Immunol..

[2] Crawford A., Angelosanto J.M., Kao C., Doering T.A., Odorizzi P.M., Barnett B.E., Wherry E.J. (2014). Molecular and Transcriptional Basis of Cd4(+) T Cell Dysfunction During Chronic Infection. Immunity.

[3] D’Souza M., Fontenot A.P., Mack D.G., Lozupone C., Dillon S., Meditz A., Wilson C.C., Connick E., Palmer B.E. (2007). Programmed Death 1 Expression on Hiv-Specific Cd4+ T Cells Is Driven by Viral Replication and Associated with T Cell Dysfunction. J. Immunol..

[4] Day C.L., Kaufmann D.E., Kiepiela P., Brown J.A., Moodley E.S., Reddy S., Mackey E.W., Miller J.D., Leslie A.J., DePierres C. (2006). Pd-1 Expression on Hiv-Specific T Cells Is Associated with T-Cell Exhaustion and Disease Progression. Nature.

[5] Haase A.T., Henry K., Zupancic M., Sedgewick G., Faust R.A., Melroe H., Cavert W., Gebhard K., Staskus K., Zhang Z.Q. (1996). Quantitative Image Analysis of Hiv-1 Infection in Lymphoid Tissue. Science.

[6] Jones L.E., Perelson A.S. (2007). Transient Viremia, Plasma Viral Load, and Reservoir Replenishment in Hiv-Infected Patients on Antiretroviral Therapy. J. Acquir. Immune. Defic. Syndr..

[7] Nakanjako D., Ssewanyana I., Mayanja-Kizza H., Kiragga A., Colebunders R., Manabe Y.C., Nabatanzi R., Kamya M.R., Cao H. (2001). High T-Cell Immune Activation and Immune Exhaustion among Individuals with Suboptimal Cd4 Recovery after 4 Years of Antiretroviral Therapy in an African Cohort. BMC Infect. Dis.

[8] Pallikkuth S., Fischl M.A., Pahwa S. (2013). Combination Antiretroviral Therapy with Raltegravir Leads to Rapid Immunologic Reconstitution in Treatment-Naive Patients with Chronic Hiv Infection. J. Infect. Dis..

[9] Serrano-Villar S., Sainz T., Lee S.A., Hunt P.W., Sinclair E., Shacklett B.L., Ferre A.L., Hayes T.L., Somsouk M., Hsue P.Y. (2014). Hiv-Infected Individuals with Low Cd4/Cd8 Ratio Despite Effective Antiretroviral Therapy Exhibit Altered T Cell Subsets, Heightened Cd8+ T Cell Activation, and Increased Risk of Non-Aids Morbidity and Mortality. PLoS Pathog..

[10] Grabmeier-Pfistershammer K., Steinberger P., Rieger A., Leitner J., Kohrgruber N. (2011). Identification of Pd-1 as a Unique Marker for Failing Immune Reconstitution in Hiv-1-Infected Patients on Treatment. J. Acquir. Immune. Defic. Syndr..

[11] Hurst J., Hoffmann M., Pace M., Williams J.P., Thornhill J., Hamlyn E., Meyerowitz J., Willberg C., Koelsch K.K., Robinson N. (2015). Immunological Biomarkers Predict Hiv-1 Viral Rebound after Treatment Interruption. Nat. Commun..

[12] Thomas A., Hammarlund E., Gao L., Holman S., Michel K.G., Glesby M., Villacres M.C., Golub E.T., Roan N.R., French A.L. (2020). Loss of Preexisting Immunological Memory among Human Immunodeficiency Virus-Infected Women Despite Immune Reconstitution with Antiretroviral Therapy. J. Infect. Dis..

[13] Elrefaei M., McElroy M.D., Preas C.P., Hoh R., Deeks S., Martin J., Cao H. (2004). Central Memory Cd4+ T Cell Responses in Chronic Hiv Infection Are Not Restored by Antiretroviral Therapy. J. Immunol..

[14] Puissant-Lubrano B., Combadiere B., Duffy D., Wincker N., Frachette M.J., Ait-Mohand H., Verrier B., Katlama C., Autran B. (2009). Influence of Antigen Exposure on the Loss of Long-Term Memory to Childhood Vaccines in Hiv-Infected Patients. Vaccine.

[15] Burton C.T., Goodall R.L., Samri A., Autran B., Kelleher A.D., Poli G., Pantaleo G., Gotch F.M., Imami N., Initio Trial International Co-ordinating Committee (2008). Restoration of Anti-Tetanus Toxoid Responses in Patients Initiating Highly Active Antiretroviral Therapy with or without a Boost Immunization: An Initio Substudy. Clin. Exp. Immunol..

[16] Hardy G.A., Imami N., Sullivan A.K., Pires A., Burton C.T., Nelson M.R., Gazzard B.G., Gotch F.M. (2003). Reconstitution of Cd4+ T Cell Responses in Hiv-1 Infected Individuals Initiating Highly Active Antiretroviral Therapy (Haart) Is Associated with Renewed Interleukin-2 Production and Responsiveness. Clin. Exp. Immunol..

[17] Wendland T., Furrer H., Vernazza P.L., Frutig K., Christen A., Matter L., Malinverni R., Pichler W.J. (1999). Haart in Hiv-Infected Patients: Restoration of Antigen-Specific Cd4 T-Cell Responses in Vitro Is Correlated with Cd4 Memory T-Cell Reconstitution, Whereas Improvement in Delayed Type Hypersensitivity Is Related to a Decrease in Viraemia. AIDS.

[18] Gay C.L., Bosch R.J., Ritz J., Hataye J.M., Aga E., Tressler R.L., Mason S.W., Hwang C.K., Grasela D.M., Ray N. (2017). Clinical Trial of the Anti-Pd-L1 Antibody Bms-936559 in Hiv-1 Infected Participants on Suppressive Antiretroviral Therapy. J. Infect. Dis..

[19] Geretti A.M., Doyle T. (2010). Immunization for Hiv-Positive Individuals. Curr. Opin. Infect. Dis..

[20] Kerneis S., Launay O., Turbelin C., Batteux F., Hanslik T., Boelle P.Y. (2014). Long-Term Immune Responses to Vaccination in Hiv-Infected Patients: A Systematic Review and Meta-Analysis. Clin. Infect. Dis..

[21] Barber D.L., Wherry E.J., Masopust D., Zhu B., Allison J.P., Sharpe A.H., Freeman G.J., Ahmed R. (2006). Restoring Function in Exhausted Cd8 T Cells During Chronic Viral Infection. Nature.

[22] Fuller M.J., Callendret B., Zhu B., Freeman G.J., Hasselschwert D.L., Satterfield W., Sharpe A.H., Dustin L.B., Rice C.M., Grakoui A. (2013). Immunotherapy of Chronic Hepatitis C Virus Infection with Antibodies against Programmed Cell Death-1 (Pd-1). Proc. Natl. Acad. Sci. USA.

[23] Anthony K.B., Yoder C., Metcalf J.A., DerSimonian R., Orenstein J.M., Stevens R.A., Falloon J., Polis M.A., Lane H.C., Sereti I. (2003). Incomplete Cd4 T Cell Recovery in Hiv-1 Infection after 12 Months of Highly Active Antiretroviral Therapy Is Associated with Ongoing Increased Cd4 T Cell Activation and Turnover. J. Acquir. Immune. Defic. Syndr..

[24] Yokosuka T., Takamatsu M., Kobayashi-Imanishi W., Hashimoto-Tane A., Azuma M., Saito T. (2012). Programmed Cell Death 1 Forms Negative Costimulatory Microclusters That Directly Inhibit T Cell Receptor Signaling by Recruiting Phosphatase Shp2. J. Exp. Med..

[25] Ghoneim H.E., Fan Y., Moustaki A., Abdelsamed H.A., Dash P., Dogra P., Carter R., Awad W., Neale G., Thomas P.G. (2017). De Novo Epigenetic Programs Inhibit Pd-1 Blockade-Mediated T Cell Rejuvenation. Cell.

[26] Pauken K.E., Sammons M.A., Odorizzi P.M., Manne S., Godec J., Khan O., Drake A.M., Chen Z., Sen D.R., Kurachi M. (2016). Epigenetic Stability of Exhausted T Cells Limits Durability of Reinvigoration by Pd-1 Blockade. Science.

[27] Wang T.W., Johmura Y., Suzuki N., Omori S., Migita T., Yamaguchi K., Hatakeyama S., Yamazaki S., Shimizu E., Imoto S. (2022). Blocking Pd-L1-Pd-1 Improves Senescence Surveillance and Ageing Phenotypes. Nature.

[28] De Sousa Linhares A., Battin C., Jutz S., Leitner J., Hafner C., Tobias J., Wiedermann U., Kundi M., Zlabinger G.J., Grabmeier-Pfistershammer K. (2019). Therapeutic Pd-L1 Antibodies Are More Effective Than Pd-1 Antibodies in Blocking Pd-1/Pd-L1 Signaling. Sci. Rep..

[29] Butte M.J., Keir M.E., Phamduy T.B., Sharpe A.H., Freeman G.J. (2007). Programmed Death-1 Ligand 1 Interacts Specifically with the B7-1 Costimulatory Molecule to Inhibit T Cell Responses. Immunity.

[30] Chan Y.T., Cheong H.C., Tang T.F., Rajasuriar R., Cheng K.K., Looi C.Y., Wong W.F., Kamarulzaman A. (2022). Immune Checkpoint Molecules and Glucose Metabolism in Hiv-Induced T Cell Exhaustion. Biomedicines.

[31] Blackburn S.D., Shin H., Haining W.N., Zou T., Workman C.J., Polley A., Betts M.R., Freeman G.J., Vignali D.A., Wherry E.J. (2009). Coregulation of Cd8+ T Cell Exhaustion by Multiple Inhibitory Receptors During Chronic Viral Infection. Nat. Immunol..

[32] Chew G.M., Fujita T., Webb G.M., Burwitz B.J., Wu H.L., Reed J.S., Hammond K.B., Clayton K.L., Ishii N., Abdel-Mohsen M. (2016). Tigit Marks Exhausted T Cells, Correlates with Disease Progression, and Serves as a Target for Immune Restoration in Hiv and Siv Infection. PLoS Pathog..

[33] Malmberg R., Zietse M., Dumoulin D.W., Hendrikx J., Aerts J., van der Veldt A.A.M., Koch B.C.P., Sleijfer S., van Leeuwen R.W.F. (2022). Alternative Dosing Strategies for Immune Checkpoint Inhibitors to Improve Cost-Effectiveness: A Special Focus on Nivolumab and Pembrolizumab. Lancet. Oncol..

[34] Wang D.Y., Salem J.E., Cohen J.V., Chandra S., Menzer C., Ye F., Zhao S., Das S., Beckermann K.E., Ha L. (2018). Fatal Toxic Effects Associated with Immune Checkpoint Inhibitors: A Systematic Review and Meta-Analysis. JAMA Oncol..

[35] Osuch S., Metzner K.J., Cortes K.C. (2020). Reversal of T Cell Exhaustion in Chronic Hcv Infection. Viruses.

